# Diamond-like carbon coating to inner surface of polyurethane tube reduces *Staphylococcus aureus* bacterial adhesion and biofilm formation

**DOI:** 10.1007/s10047-023-01403-1

**Published:** 2023-05-25

**Authors:** Noriaki Kuwada, Yasuhiro Fujii, Tatsuyuki Nakatani, Daiki Ousaka, Tatsunori Tsuji, Yuichi Imai, Yasuyuki Kobayashi, Susumu Oozawa, Shingo Kasahara, Kazuo Tanemoto

**Affiliations:** 1 Department of Cardiovascular Surgery, Kawasaki Medical School, 577 Matsushima, Kurashiki-City, Okayama 701-0192 Japan; 2 Department of Cardiovascular Surgery, Okayama University Faculty of Medicine, Dentistry and Pharmaceutical Sciences, 2-5-1 Shikata-Cho, Kita-Ku, Okayama-City, Okayama 700-8558 Japan; 3 Institute of Frontier Science and Technology, Okayama University of Science, 1-1 Ridai-Cho, Kita-Ku, Okayama-City, Okayama Japan; 4 Department of Pharmacology, Okayama University Faculty of Medicine, Dentistry and Pharmaceutical Sciences, 2-5-1 Shikata-Cho, Kita-Ku, Okayama-City, Okayama 700-8558 Japan; 5 Division of Cardiovascular Surgery, Department of Surgery, Labatt Family Heart Centre, The Hospital for Sick Children, University of Toronto, 555 University Avenue, Toronto, ON M5G 1X8 Canada; 6 Division of Medical Safety Management, Safety Management Facility, Okayama University Hospital, 2-5-1 Shikata-Cho, Kita-Ku, Okayama-City, Okayama 700-8558 Japan

**Keywords:** Diamond-like carbon, Polyurethanes, Luminal coating, Staphylococcus aureus, Prevention of infection

## Abstract

*Staphylococcus aureus* is one of the main causative bacteria for polyurethane catheter and artificial graft infection. Recently, we developed a unique technique for coating diamond-like carbon (DLC) inside the luminal resin structure of polyurethane tubes. This study aimed to elucidate the infection-preventing effects of diamond-like carbon (DLC) coating on a polyurethane surface against *S. aureus*. We applied DLC to polyurethane tubes and rolled polyurethane sheets with our newly developed DLC coating technique for resin tubes. The DLC-coated and uncoated polyurethane surfaces were tested in smoothness, hydrophilicity, zeta-potential, and anti-bacterial properties against *S. aureus* (biofilm formation and bacterial attachment) by contact with bacterial fluids under static and flow conditions. The DLC-coated polyurethane surface was significantly smoother, more hydrophilic, and had a more negative zeta-potential than did the uncoated polyurethane surface. Upon exposure to bacterial fluid under both static and flow conditions, DLC-coated polyurethane exhibited significantly less biofilm formation than uncoated polyurethane, based on absorbance measurements. In addition, the adherence of *S. aureus* was significantly lower for DLC-coated polyurethane than for uncoated polyurethane under both conditions, based on scanning electron microscopy. These results show that applying DLC coating to the luminal resin of polyurethane tubes may impart antimicrobial effects against *S. aureus* to implantable medical polyurethane devices, such as vascular grafts and central venous catheters.

## Introduction

Polyurethane is widely used in several medical devices that is implanted in the body, such as in drip tubes, indwelling needles, central venous catheters, and artificial blood vessels, owing to its high biocompatibility, low biodegradability, and appropriate bending resistance [1] However, polyurethane artificial blood vessels are more vulnerable to infection than extended polytetrafluoroethylene (ePTFE) [2, 3]. Therefore, polyurethane’s susceptibility to infection is an important issue that requires attention.

Diamond-like carbon (DLC) films are amorphous carbon-based films containing intermingled *sp*^*3*^ and *sp*^*2*^ carbon–carbon bonds. They are known for their high biocompatibility [4], hardness, low friction coefficients, chemical inertness, and smooth surface finish [5]. DLC was originally used for metals; however, it is now used as a resin coating because of recent developments in coating technology. Recently, we have developed a unique technique for coating DLC inside the luminal structure of resin tubes DLC using an alternative current high-voltage methane plasma chemical vapor deposition method with an originally developed DLC coating system [6].

*Staphylococcus aureus* is one of the main causative bacteria of catheter and artificial graft infections [7]. Previous studies have shown that DLC coating decreases *S. aureus* attachment to the surface of ultra-high molecular weight polyethylene [8] and inhibit colony formation on silicon surfaces [9, 10]. In addition, several reports describe the preventative effects of DLC against various bacteria infections, with or without a doping substance [11–17]. Better clinical results for catheters or artificial graft implantation could be achieved if a DLC coating for the inner surface of resin tubes could be developed to prevent *S. aureus* infection; however, this has not yet been investigated to date. Therefore, our study aimed to elucidate the effects of DLC coatings on polyurethane surfaces in preventing *S. aureus* infection*.*

## Materials and methods

### DLC coatings for polyurethane tubes and sheets

Polyurethane tubes (5 mm diameter, Uxcell, Hong Kong, China) and sheets (0.15 mm thickness, JINYANG URETHANE CO., LTD, Seoul, Korea) were first washed with 70% ethanol to prepare the coating. The internal surfaces of the 5 mm polyurethane tubes were coated with DLC using an alternative current high-voltage methane plasma chemical vapor deposition method [6]. The interior chamber of this system was adjusted to < 7 × 10^−3^ Pa. Methane gas was used as the source gas, with a flow rate of 96 standard cubic cm/min using a mass flow controller. The operational pressure was 39 Pa with a constant AC voltage of 5 kV and a frequency of 10 kHz. The coating duration was 10 min. Regarding the coating of the polyurethane sheet, the sheet is rolled into a cylindrical shape, placed in a coating apparatus, and coated under the same conditions. Therefore, the coating methods of polyurethane tubes and polyurethane sheets are equivalent. The existence of coated DLC was confirmed by Raman spectroscopy as described previously [6].

### Surface roughness measurement

Nine 1-cm-square pieces of DLC-coated and uncoated polyurethane sheets were used because plane surface is required for the accurate measurement. The surface roughness of the DLC-coated and uncoated polyurethane sheet surfaces was quantified by 3D non-contact surface scanning using a New View 5320 scanner (Zygo Corporation, Middlefield, OH, USA) and expressed as maximum profile valley depth (PV), root mean square deviation (Sq), and arithmetical mean height (Sa).

### Water contact angle measurement

Ten 1 cm^2^ pieces of DLC-coated and uncoated polyurethane sheets were used because a planar surface is required for the accurate measurement. The hydrophilicity of the DLC-coated and uncoated polyurethane sheets was assessed by measuring the static contact angle between a 2 μL deionized water drop and the surface of each sheet using DropMaster500 (Kyowa Interface Science, Saitama, Japan).

### Zeta-potential measurement

Three pairs of flat DLC-coated and uncoated polyurethane sheets were used to measure the zeta-potential because a planar surface was required for the accurate measurement which were obtained using an ELSZ-1000 analyzer (Otsuka Electronics Co., Ltd., Osaka, Japan). The zeta-potential of the surface of a solid sample was determined by measuring the electric mobility at different points in a flat cell of the ELSZ-1000 and analyzing the electroosmotic flow using the Mori–Okamoto equation [18]. A measurement sample of 25 mm × 7 mm was set in the plating cell, and 100 mL distilled water and 500 μL monitoring particles (Otsuka Electronics Co., Ltd., Osaka, Japan) were adjusted for measurement. The polyurethane sheet was attached to a chamber of the ELSZ-1000, and the chamber was filled with a 10 mM NaCl solution, where monitoring particles were suspended. Then, electrophoresis of the particles was conducted, and the apparent velocity distribution in the chamber was determined. The electrophoresis conditions were as follows: mean electric field intensity, 17.33 V/cm, and average current, 1.02 mA. The measurement was performed three times for three each sample and averaged in DLC-coated and uncoated polyurethane sheet.

### Bacterial strains and culture conditions

A clinically isolated *S. aureus* strain (ATCC 29213) was used because of its high biofilm-forming capacity. Frozen stock of the *S. aureus* strain was grown overnight in 10 mL Luria–Bertani broth (Nacalai Tesque, Kyoto, Japan) at 37 °C. It was then diluted 1:100 with a fresh medium supplemented with magnesium sulfate (0.246 g), 0.2% glucose (0.2 g), 0.4% casamino acid (0.4 g), and 100 mL of normal saline. The *S. aureus* was then incubated overnight at 37 °C with agitation. This fresh medium-diluted mixture was used for static and perfusion conditions because it is less prone to planktonic growth and creates a more robust biofilm [19].

### Exposure to *S. aureus* under static condition

Three pairs of DLC-coated and uncoated polyurethane tubes were used. Five 1-cm-length tubes were created from each DLC-coated or uncoated polyurethane tube for this study. These tubes were placed vertically in microwell plates with 1500 μL of bacterial solution and incubated at 37 °C for 24 h with agitation at 60 beats/min, using a WAVE-SI shaking system (TAITEC, Saitama, Japan). In all tubes, 0.75 cm out of the 1-cm-length was immersed in the bacterial solution. Four of the five 1-cm-length tubes from a tube were used for biofilm quantification and the remaining 1-cm-length tube was used for scanning electron microscope (SEM) observation.

### Exposure to *S. aureus* under flow condition

**Fig. 1:**
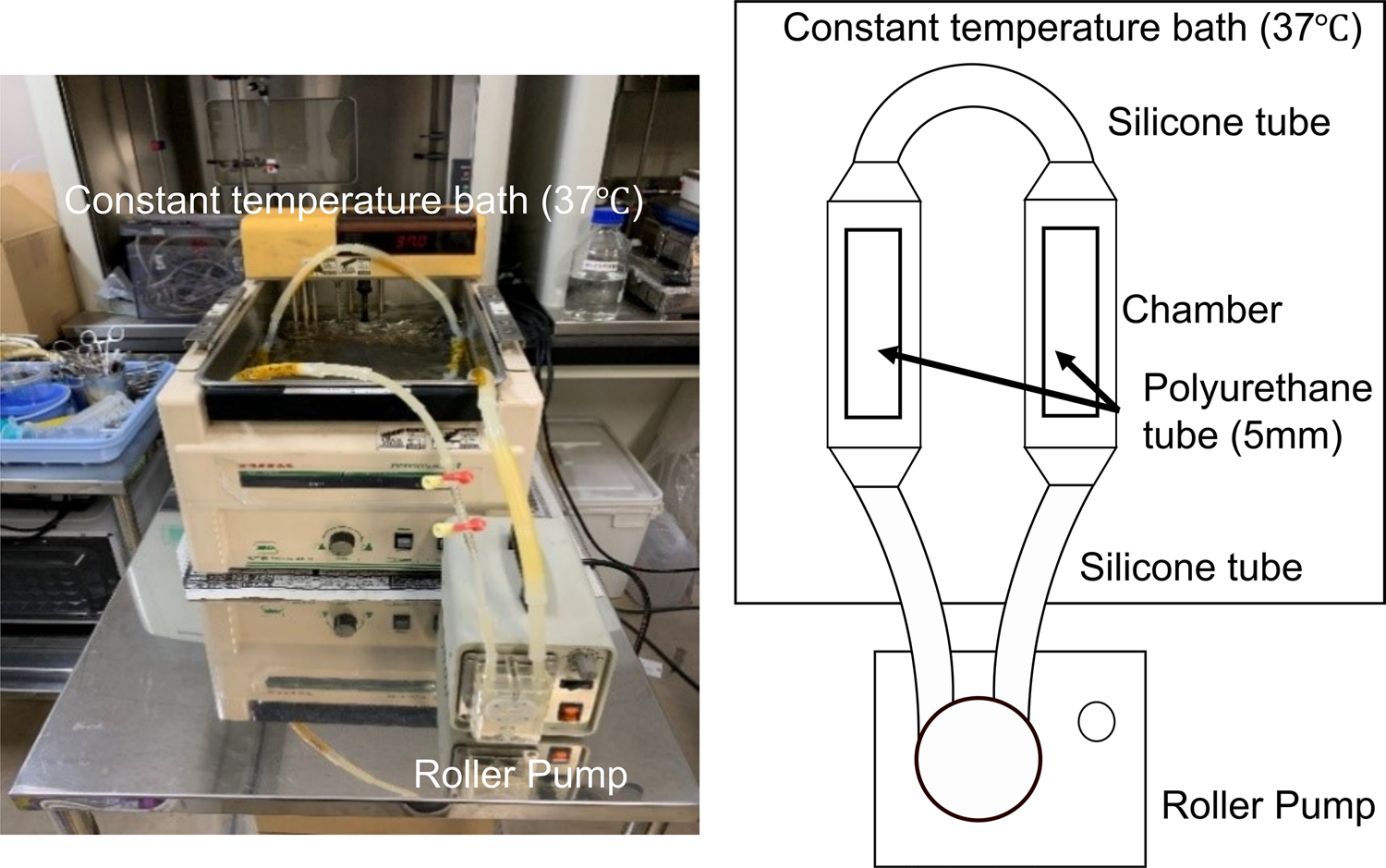
Circuit for perfusion condition. A pair of DLC-coated and uncoated polyurethane tubes were placed in the chambers of the silicone tube circuit. The bacterial fluid was perfused in the circuit to expose the surface of the polyurethane tube to the bacteria. The bacterial fluid was supplied at a flow rate of 60 mL/min using a roller pump. The silicone chambers were immersed in a constant-temperature bath at 37 °C

Two pairs of 5-cm DLC-coated and uncoated polyurethane tubes were used. The continuous-flow circuit used in this experiment is shown in Fig. 1. A pair of DLC-coated and uncoated polyurethane tubes were placed in the chambers of the silicone tube circuit. The bacterial fluid was perfused in the circuit exposing the luminal surface of the polyurethane tube to the bacterial fluid. The bacterial fluid was supplied at a flow rate of 60 mL/min using a roller pump. The silicone chambers were immersed in 37 °C a constant-temperature bath. Each polyurethane tube was divided and used for biofilm quantification and SEM observations.

### Quantification of the biofilm with absorbance measurement

Biofilm formation by *S. aureus* was evaluated on crystal violet staining as described previously [19]. Polyurethane tubes obtained from the static and flow conditions were then lightly washed with saline, immersed into 2000 μL of 0.1% crystal violet solution for 10–15 min and washed lightly again with saline. They were then immersed in 2000 μL of 30% acetic acid for 30 min to remove the adsorbed crystal violet stain. The absorbance of the stain was measured using a Promega detection system (GloMax® Multi+, Sunnyvale, USA) with 560 nm wavelength.

### Bacterial adhesion assays (SEM observation)

The bacterial adhesion assays consisted of counting bacteria and visualizing the adhered bacterial cells using SEM in the DLC-coated and uncoated polyurethane tubes. The polyurethane tubes from the static and flow conditions (three tubes and two tubes from each group, respectively) were fixed with 10% formaldehyde in phosphate-buffered saline overnight at 4 °C. They were post-fixed in 1% osmium tetroxide (OsO_4_) and dehydrated using an ethanol series. The tubes were critically dried, cut in half in the sagittal direction, mounted on stubs lumen side up for observation, and deposited in OsO_4_. The tubes were mounted in the SEM to obtain micrographs using a HITACHI S-4800 SEM (Hitachi High-Tech Corporation, Tokyo, Japan). The number of attached bacteria in each tube was counted in 10 randomly selected fields of view at a magnification of 2000× and averaged.

### Statistics

All data were analyzed using SPSS Statistics version 28 (IBM Corp, Armonk, NY, USA). Differences in the number of bacterial counts and absorbance between the DLC-coated and uncoated polyurethane tubes were evaluated using a paired *t*-test. All *P*-values were two-sided, and a *P*-value < 0.05 indicates statistical significance. Continuous values are represented as mean ± standard deviation.

## Results

### Raman spectroscopy

**Fig. 2:**
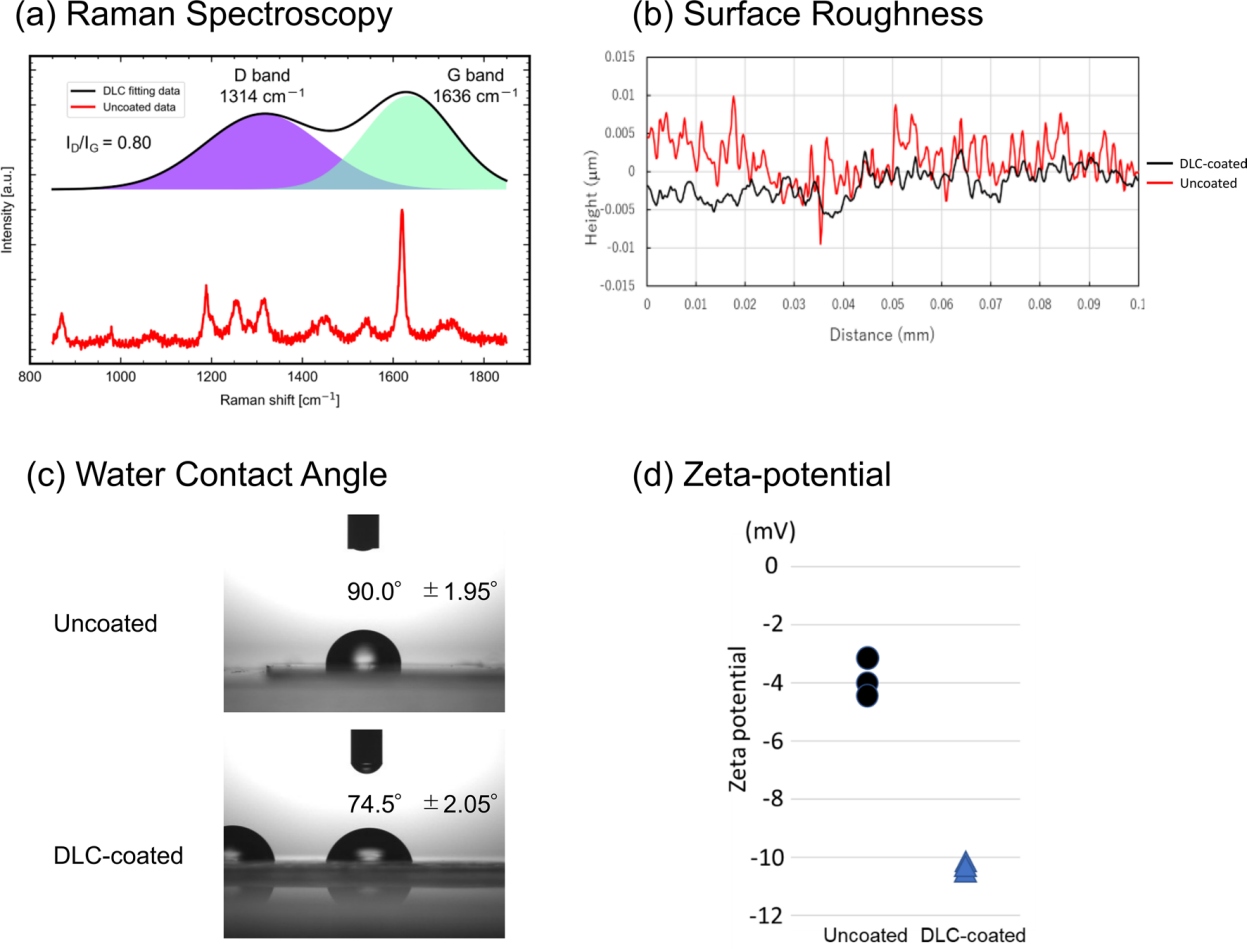
Comparison of surface properties between DLC-coated and uncoated polyurethane surface: **a** results of Raman spectroscopy, **b** results of surface roughness measurements; PU, polyurethane, **c** results of water contact angle, and **d** results of zeta-potential measurements

The results of Raman spectroscopy are shown in Fig. 2a. The black and red lines express the fitting data of DLC-coated polyurethane with Voigt function and raw data of uncoated polyurethane, respectively. A large peak characteristic of polyurethane was observed near 1619 cm^−1^ on the uncoated polyurethane surface [20]. The intensity was increased on the surface of DLC-coated polyurethane from uncoated polyurethane. The success of the DLC coating was confirmed with typical D and G bands. Visible Raman spectra of DLC are dominated by scattering from *sp*^*2*^ the sites. The spectra typically have two main peaks, the D peak around 1350 cm and the G peak around 1580 cm which correspond to the breathing mode of the aromatic rings and the *sp*^*2*^ stretching mode of pairs of sites in the aromatic rings or the olefinic chains [5, 21–23].

### Surface roughness

Figure 2b shows an example of the surface roughness measurements. The statistical comparison of the DLC-coated and uncoated polyurethane surface showed that PV was significantly smaller (13.7 ± 4.2 nm [range 10.0–22.0 nm] vs. 18.3 ± 3.3 nm [range 14.0–25.0 nm] vs, *P* = 0.006], the Sq was significantly smaller (2.2 ± 0.4 nm [range 2.0–3.0 nm] vs. 4.0 ± 1.3 nm [range 3.0–7.0 nm], *P* = 0.007), and the Sa was significantly smaller (1.9 ± 0.3 nm [range, 1.0–2.0 nm] vs. 3.2 ± 1.1 nm [range, 2.0–6.0 nm], *P* = 0.01) in DLC-coated polyurethane surface than in uncoated polyurethane surface. These results indicate that the DLC-coated polyurethane surface was significantly smoother than uncoated polyurethane surface. The DLC-coated surface exhibit 25% decrease in PV, 45% decrease in Sq, and 41% decrease in Sa.

### Water contact angle

The water contact angle was significantly lower for DLC-coated polyurethane than for uncoated polyurethane (74.5° ± 2.05° [range 71.6°–78.7°] vs. 90.0° ± 1.95° [range 86.3°–92.4°], *P* < 0.001), confirming an increased hydrophilicity (Fig. 2c).

### Zeta-potential

The zeta-potential values were significantly lower in DLC-coated polyurethane than uncoated polyurethane (−10.21 mV, −10.30 mV, and −10.66 mV in DLC-coated polyurethane vs. −3.14 mV, −4.10 mV, and −4.37 mV in uncoated polyurethane, *P* = 0.018) (Fig. 2d).

### Biofilm formation and bacterial attachment under static condition

**Fig. 3:**
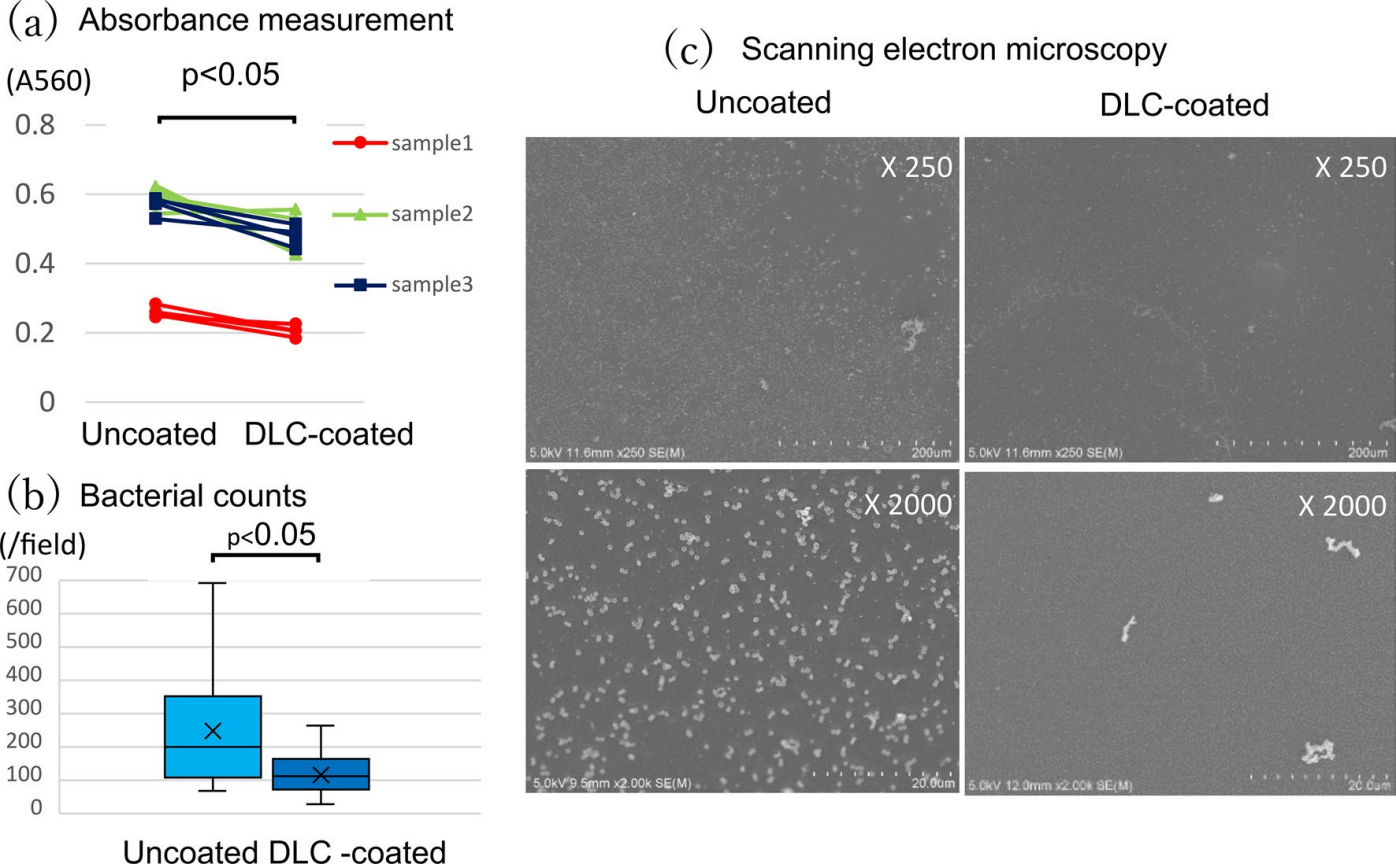
Results of the static condition: **a** results of absorbance measurement for biofilm, **b** results of attached bacteria counts, **c** scanning electron microscope analysis of DLC-coated and uncoated polyurethane surfaces

In the static condition, absorbance measurements with crystal violet staining showed that DLC-coated polyurethane significantly suppressed biofilm formation when compared with uncoated polyurethane (0.392 ± 0.142 [range 0.187–0.557] vs. 0.475 ± 0.160 [range 0.248–0.625], *P* < 0.001) (Fig. 3a). The proportion of attached bacteria was significantly lower in DLC-coated polyurethane than in uncoated polyurethane (118 ± 60/field [range 40–264/field] vs. 250 ± 166/field [range 68–693/field], *P* < 0.001) (Fig. 3b). SEM observation showed that *S. aureus* adhesion was suppressed in DLC-coated polyurethane compared with uncoated polyurethane (Fig. 3c).

### Biofilm formation and bacterial attachment under flow condition

**Fig. 4:**
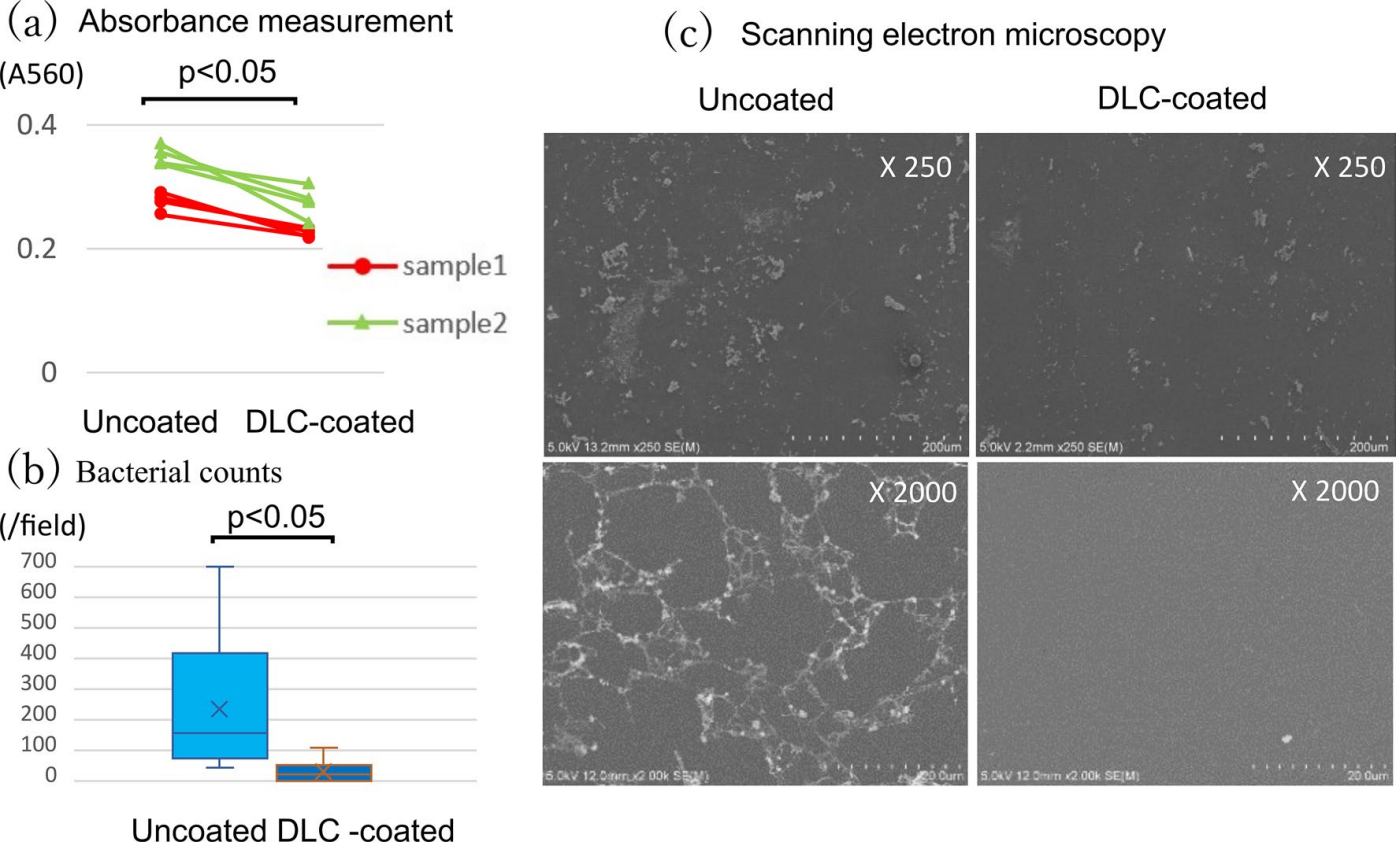
Results of perfusion condition: **a** results of absorbance measurement for biofilm, **b** results of attached bacteria counts, **c** scanning electron microscope analysis of DLC-coated and uncoated polyurethane surfaces

In the flow condition, DLC-coated polyurethane significantly suppressed biofilm when compared with uncoated polyurethane (0.250 ± 0.033 [range 0.218–0.305] vs. 0.313 ± 0.042 [range 0.256–0.370], *P* < 0.001) (Fig. 4a). The proportion of attached bacteria was significantly lower in DLC-coated polyurethane than in uncoated polyurethane (31 ± 8/field [range 0–110/field] vs. 236 ± 44/field [range 43–700/field], *P* < 0.001) (Fig. 4b). SEM observation showed that *S. aureus* adhesion was suppressed in DLC-coated polyurethane compared with uncoated polyurethane (Fig. 4c).

## Discussion

Some reports have described the anti-bacterial effects of DLC-based hybrid or metal atom-doped coating against *S. aureus* [11–13, 24]. However, as far as we searched, there are only two studies describing the anti-bacterial effects of DLC by itself against *S. aureus*. Del Prado et al. showed that DLC coating decreased *S. aureus* attachment on the surface of ultra-high molecular weight polyethylene (UHMWPE) [8]. Levon and Myllymaa et al. showed that DLC coating inhibited *S. aureus* colony and biofilm formation on silicon surfaces [9, 10]. However, no studies have yet reported the effects of DLC coating of polyurethane surfaces in preventing *S. aureus* adhesion and biofilm formation. In this study, DLC coating was proven to suppress the adhesion and biofilm formation of *S. aureus* on polyurethane surface as demonstrated with UHMWPE and silicon.

Previously, we reported that DLC coating made by the same method as in this study inhibited the adhesion of *Pseudomonas aeruginosa* and *Escherichia coli*, as well as the biofilm formation of *P. aeruginosa* on the inner surface of the silicon tube under flow conditions with artificial urine. However, these effects could not be obtained against *S. aureus* [14]. This is probably because artificial urine was used as the culture medium and the substrate also differed from this study. The differences of flow condition and length of incubation time also might affect the outcomes. This kind of variability in results is seen in studies investigating the antimicrobial properties of DLC against *Candida albicans*, whether it is polyurethane-based or silicone-based [25, 26]. Establishing protocols to evaluate anti-infective properties according to use will be important for accelerating the development of antimicrobial coatings for implantable medical devices.

This study showed decreased adhesion and biofilm formation of *S. aureus* using DLC-coated polyurethane tubes, compared with uncoated polyurethane tubes. These effects were observed under both static and perfused conditions, to replicate exposure to the human bloodstream. These kinds of antibacterial tests are usually performed under static conditions; however, in the static condition, there is a difference in bacterial concentration between the deep and surface layers, and the air may affect the bacteria activity and the ability of bacteria to attach to the testing material surface [19]. In addition, solution flow may affect bacterial adhesion under the perfused conditions [24, 27]. As we envisioned that this coating will be in contact with the bloodstream, we added the experiment assessing flow conditions. Subsequently, the results of the flow studies reinforced the validity of the results under static conditions.

The physical properties of polyurethane surface were significantly changed by DLC coating. The DLC-coated polyurethane surface had a significantly decreased water contact angle, smoother surface, and a lower zeta-potential than the uncoated surface. The decreased water contact angle from 90.0° to 74.5° indicates increased hydrophilicity and surface free energy. Generally, a surface with moderate hydrophilicity increases bacterial attachment [28, 29]. Yuan et al. described that a water contact angle of 90° was the least optimal for bacterial attachment prevention [30]. The increased hydrophilicity on DLC-coated polyurethane tube reduced the *S. aureus* adhesion. In addition, DLC-coated polyurethane surface was smoother than that of the uncoated surface, as determined by calculating the PV, Sq, and Sa values. Increased smoothness is associated with low friction [17], whereas a larger surface area provides more area for bacterial adherence, eventually supporting bacterial growth [31–34]. Because of this, it is possible that the increased surface smoothness suppresses the adhesion of *S. aureus*. Furthermore, the zeta-potential of DLC-coated polyurethane was lower than uncoated polyurethane. In general, for most bacteria, the net surface charge is negative and balanced by oppositely charged counter-ions present in the surrounding media [35]. In fact, the average zeta-potential of the untreated *S. aureus* was found to be − 35.6 mV [36]. DLC-coated polyurethane may prevent bacterial adhesion because of the repelling effect of the more negative zeta-potential on the negatively charged *S. aureus*. Although the individual effects may not be conclusive, it is likely that the combination of these beneficial properties of DLC-coated surfaces facilitate the inhibition of *S. aureus* adhesion and biofilm formation.

Clinically, polyurethane is often used for catheters and vascular grafts. Polyurethane and ePTFE are both the main biomaterials as vascular grafts for hemodialysis; therefore, they have sometimes compared the features in this function. Polyurethane vascular grafts are reportedly more vulnerable to bacterial infection than ePTFE artificial blood vessels [2.3]. Thus, the usefulness of polyurethane for catheter and vascular prostheses would increase with improved antimicrobial properties. As *S. aureus* is one of the most prominent bacterial species in catheters or artificial vascular graft-related infections [7], DLC coating could prove beneficial. In addition, DLC can be used in metallic parts of blood contact devices [37, 38]; therefore, the DLC for resin tubes is also expected to be bio-compatible enough to be used in artificial blood vessels and catheters. We are currently conducting a demonstration experiment on this topic.

Current thought is that the effects of DLC coating will change depending on the properties and steric structure of the substrate. Thus, detailed investigations according to use are indicated for each combination of material and bacteria to confirm the effects of DLC. Further study will be necessary to determine if these results have clinical significance. However, as DLC itself is non-toxic and hypoallergenic for humans, DLC may prove superior in terms of safety for developing new coatings to enhance the clinical performance of biomaterials.

### Study limitation

This study has six main limitations. First, the number of polyurethane tubes in each group was small. However, the results of this experiment were largely determined by the engineering conditions under which the DLC was made which can be strictly controlled. In addition, we used the paired comparisons for each experiment and obtained highly reproducible results. Second, as the concentrations of prepared bacterial solutions were not measured, they may have differed between experiments. To minimize this limitation, we used the same bacterial solution for each paired comparison of DLC-coated and uncoated; therefore, this limitation is not anticipated to affect the results substantially. Third, we performed in vitro studies only; *S. aureus* attachment or biofilm formation may behave differently in vivo. Future in vivo studies are warranted before commercialization of DLC coatings. Fourth, the perfusion studies used steady flow rather than pulsatile flow. Bacterial attachment and biofilm formation in pulsatile flow conditions might differ from those in continuous-flow conditions. Fifth, under flow conditions, the outer surface of the tube not coated with DLC is in contact with the bacterial solution, so the difference in absorbance values for biofilm measurements should be underestimated. However, this limitation does not affect the outcome. Finally, the DLC-coated sheet was used to verify the water contact angle, smoothness, and zeta-potential because the flat surface was needed for these examinations. As the DLC coating method for the polyurethane sheets was the same as that of DLC-coated tubes, the influence of this limitation on the results should be minimal because if the structure of DLC is the same, even if the steric structure of the substrate changes, it is expected that the direction of change will be the same.

## Conclusion

DLC coating on the inner surface of polyurethane tubes inhibited the adhesion and biofilm formation of *S. aureus*. These results indicate that applying DLC coating will provide anti-bacterial protection against *S. aureus* to implantable polyurethane medical tubular devices such as vascular grafts and central venous catheters.

## Data Availability

The datasets generated and/or analyzed during the current study are available from the corresponding author on reasonable request.
